# Detection of Antimicrobial Peptides in Stratum Corneum by Mass Spectrometry

**DOI:** 10.3390/ijms22084233

**Published:** 2021-04-19

**Authors:** Adrienn Jenei, Gergő Kalló, Zsolt Dajnoki, Krisztián Gáspár, Andrea Szegedi, Anikó Kapitány, Éva Csősz

**Affiliations:** 1 Division of Dermatological Allergology, Department of Dermatology, Faculty of Medicine, University of Debrecen, H-4032 Debrecen, Hungary; jenei.adrienn@med.unideb.hu (A.J.); dajnoki.zsolt@med.unideb.hu (Z.D.); gaspar.krisztian@med.unideb.hu (K.G.); aszegedi@med.unideb.hu (A.S.); kapitany.aniko@med.unideb.hu (A.K.); 2 Department of Dermatology, Faculty of Medicine, University of Debrecen, H-4032 Debrecen, Hungary; 3 Department of Neurosurgery, Faculty of Medicine, University of Debrecen, H-4032 Debrecen, Hungary; 4 Gyula Petrányi Doctoral School of Allergy and Clinical Immunology, University of Debrecen, H-4032 Debrecen, Hungary; 5 Proteomics Core Facility, Department of Biochemistry and Molecular Biology, Faculty of Medicine, University of Debrecen, H-4032 Debrecen, Hungary; kallo.gergo@med.unideb.hu

**Keywords:** antimicrobial peptide, tape stripping, mass spectrometry, healthy skin, stratum corneum

## Abstract

Antimicrobial and immunomodulatory peptides (AMPs) are considered as the key players in the maintenance of skin barrier functions. Here, we developed a novel approach for the examination of AMPs in the outermost layer of the epidermis, namely stratum corneum (SC). The SC sample collection by tape stripping was coupled with detection by highly specific and sensitive parallel reaction monitoring (PRM)-based mass spectrometry. We found that hexane-free processing of SC samples produced higher protein yield compared to hexane-based extraction. Of the 18 investigated peptides, 9 could be detected either in healthy or in inflamed skin specimens. Regarding the amount of S100A8, LCN2, LACRT and LYZ significant topographical differences were described among gland poor (GP), sebaceous gland rich (SGR) and apocrine gland rich (AGR) healthy skin regions. We applied a minimally invasive, reproducible approach for sampling, which can be assessed for research and diagnostic purposes and for monitoring the effectiveness of therapies in skin diseases.

## 1. Introduction

The human skin provides an interface between our body and the external environment acting not only as a physical barrier but also as antimicrobial and immune barriers [1,2,3,4]. Skin components, such as keratinocytes, mast cells, sebocytes, neutrophil granulocytes and sweat glands synthesize small, usually 12–50 amino acid long proteins that belong to the family of antimicrobial and immunomodulatory proteins (AMPs) providing a prompt response against potentially pathogenic agents [5]. These molecules are essential components of the innate immune response and play a role in supporting the homeostasis of healthy skin. They also play a role in immunomodulatory processes, including cell migration, proliferation and differentiation, production of cytokines and chemokines and the sustenance of skin barrier functions [6].

AMPs form a layer of thin deposit on the skin surface after being secreted into the stratum corneum (SC) through exocytosis of lamellar bodies [6,7,8]. Based on their production, AMPs can be classified as constitutively produced or inducible. Dermcidin (DCD), human β-defensin-1 (hBD-1), cathelicidin (LL-37), psoriasin (S100A7), lysozyme (LYZ) and loricrin (LOR) are constitutively present on healthy human skin surface and play an important role in the maintenance of skin barrier homeostasis [9]. However, inflammation disrupts the skin barrier, which is associated with changes in the levels of AMPs. Inducible AMPs such as hBD-2, S100A12, S100A15, RNase 1, 4, 5 are produced upon inflammatory signals and can promote cell migration, proliferation, differentiation, cytokine/chemokine production, and furthermore possess pro- or anti-inflammatory, immunomodulatory properties [7,10,11,12,13,14,15,16,17,18,19]. The regulation of AMP level can imply complex mechanisms, for example DCD plays a role in the regulation of microbiota on the skin surface and cannot be induced by inflammation or skin injury. On the contrary, eccrine sweat glands’ DCD production has been demonstrated to be ceased by inflammatory events [20,21,22].

The investigation of the skin’s AMP profile may have both diagnostic and predictive value. AMPs could serve as markers for assessing the stage and severity of certain skin diseases, such as LL-37 and hBD4 in atopic dermatitis (AD), S100A7 in psoriasis vulgaris (Ps) and kallikrein 5 in papulopustular rosacea (PPR) [1,7,14,22,23]. During our work we aimed to develop a novel method to establish the AMP profile of the stratum corneum that would represent all examined conditions of the skin, under both healthy and diseased state. Accordingly, we selected AMPs, which may typically be present in either healthy or inflamed skin.

Recently, remarkable differences in microbial and chemical (sebum, sweat and pH) as well as innate and adaptive immune and permeability barrier milieu has been demonstrated on topographically distinct healthy skin areas [2,24,25]. In our previous investigations, by applying mRNA and protein based methods, we could also identify significant topographical differences regarding innate and adaptive immune and permeability barrier milieu among sebaceous and apocrine gland-poor (GP), sebaceous gland-rich (SGR) and apocrine gland-rich (AGR) healthy skin regions, and inflamed skin [2,24,25]. We also detected significantly increased expression levels of chemokines, AMPs and altered permeability barrier proteins among GP, AGR and SGR skin areas [2,25].

The examination of regional differences in healthy skin is challenging due to the lack of a quick, reliable, and minimally invasive sampling and detection technique. Current approaches for the identification of AMPs typically rely on antibody-based epitope recognition by ELISA and immunohistochemical (IHC) assays using skin biopsies. However, the limitations in specificity, and potential interference with contaminating material from the surface of the skin in the case of ELISA remain a concern. Moreover, in case of IHC assays being difficult in obtaining biopsy specimens for the measurements [21,26,27,28]. Since gaining whole skin biopsies results in scarring and pain, in our present study, we aimed to establish a minimally invasive SC sample collection process coupled with a highly reliable quantification method. This was achieved by using tape stripping followed by PRM-based mass spectrometry in order to detect, quantify, and monitor skin AMP levels under steady-state condition [29,30,31,32,33,34,35]. Parallel Reaction Monitoring (PRM) is a targeted mass spectrometry acquisition specific for hybrid tandem mass spectrometers containing a quadrupole and a high-resolution analyzer (e.g., Orbitrap) [36]. PRM analysis provides high specificity and sensitivity, allowing for the analysis of proteins with low abundance in complex biological samples [37]. The high specificity, sensitivity, the large dynamic range and the multiplex feature of PRM is highly relevant to biological applications where the amount of the samples is limited. In case of tape stripping, the amount of collected sample is low with low protein concentration, therefore the features characteristic to PRM (high sensitivity and specificity, multiplexing possibility) make PRM-based mass spectrometry a method of choice for the analysis of multiple AMPs in the same SC sample.

The overall objective of our research was to optimize a method for the rapid examination of AMP content of human skin surface and its outermost layer. To overcome the challenges of past attempts, the proteins were collected from healthy, unperturbed, uncleansed skin by adhesive tape removal.

## 2. Results

Our aim was also to determine which AMPs are characteristic to the SC in different areas of the GP, SGR and AGR skin regions of healthy individuals. During our workflow, first, we optimized a sample collection technique and PRM-based mass spectrometry method on healthy and inflamed skin. Following optimization, the level of AMPs characteristic for healthy GP, SGR and AGR skin regions was examined.

### 2.1. Method Development and Optimization for the Examination of AMPs in the Stratum Corneum

#### 2.1.1. Optimization of Sample Collection

**Figure 1:**
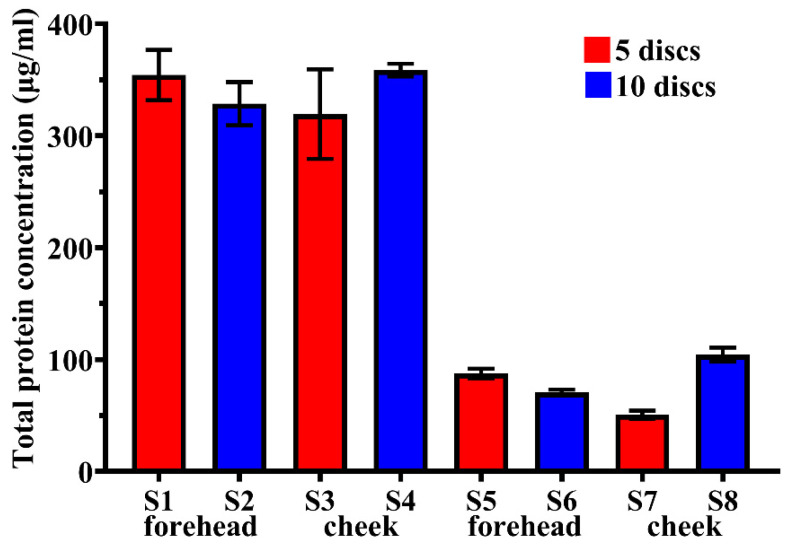
Total protein concentration of samples collected from forehead or cheek of healthy individuals. The ‘y’ axis indicates the protein concentration in samples obtained from forehead and cheek (S1–S4) of D1 volunteer and (S5–S8) of D2 volunteer shown on ‘x’ axis. The red bars represent mean ± SEM of the protein concentrations observed in samples obtained from pooling of 5 discs, while the blue bars show samples obtained from pooling of 10 discs.

The total protein amount that can be obtained from SC by D-Squame discs is limited. In order to extract the highest protein amount, we tested several sampling methods. Samples were collected from SC of healthy study subjects from the forehead and cheek. In total, 5 or 10 individual discs were pressed onto the surface of the forehead and cheek, respectively, to the same area. Proteins removed with 5 or 10 discs, respectively, were pooled and the total protein concentration was measured in each collected sample (Figure 1).

According to Figure 1, the SC total protein concentration was rather characteristic to the volunteer and not to the number of collected discs or place of collection. Taking into account that stripping the same area three or more times caused significant discomfort to volunteers, for all subsequent samplings only one disk was used for each area.

#### 2.1.2. Design and Optimization of PRM-Based Mass Spectrometry Method

The protein sequences of selected AMPs were subjected to bioinformatics analyses and the unique protein-specific tryptic peptide sequences were identified. The selected unique peptides were used for PRM analyses (Table 1).

The SIL synthetic counterparts of the selected peptides were ordered and used for quantification. The PRM data were analysed with the Skyline software and all the recorded data were uploaded to the Panorama (https://panoramaweb.org/University%20of%20Debrecen/Skin%20AMP/project-begin.view, accessed on 15 March 2021). A peptide was considered to be present in the sample when the peaks characteristic for the endogenous peptides showed coelution with the peaks characteristic for their SIL synthetic counterparts. Peaks showing no coelution with their SIL counterparts were excluded from the analyses. The integration of the spectra was performed by the software and the ratios of the endogenous and SIL peptides were determined. The calculated ratios were used for the comparative analysis of the selected AMPs in the studied groups.

In order to be sure that both constitutive and inducible AMPs can be detected from stratum corneum by PRM-based mass spectrometry method, we collected samples from volunteers with both healthy and inflamed skin. SC samples were collected from 4 patients suffering from various inflammatory skin diseases: hidradenitis suppurativa (HS), psoriasis vulgaris (Ps), papulopustular rosacea (PPR), atopic dermatitis (AD), as well as from a healthy volunteer (Table S1). HS samples were collected from armpit, Ps from the limbs, PPR from the cheek and AD from thigh, while healthy control samples were originated from the forehead region. SC samples were pooled to have a cocktail of AMPs and were used as positive control to test the PRM-based method in case of the peptides listed in Table 1.

According to the applied criteria, ALNSIIDVYHK and GADVWFK peptides from S100A8, VPLQQNFQDNQFQG peptide from LCN2, ENAGEDPGLAR peptide from DCD, SILLTEQALAK peptide from LACRT and WESGYNTR peptide from LYZ could be observed both in healthy and inflamed skin sample, while DLYNFLK peptide from S100A9, LLGDFFR peptide from LL-37 and TFVPGCQPGEFTLGNIK peptide from LCN2 were only detected in the inflamed skin sample (Table 2). The SIIGMIDMFHK peptide from S100A7, IQGTCYR peptide from hBD1, GIGDPVTCLK peptide from hBD2, GIINTLQK peptide from hBD3, ICGYGTAR peptide from hBD4, SYNVTSVLFR peptide from LCN2, CLEQVSQLQGLWR peptide from TSLP, QELNPLK peptide from LACRT and GISLANWMCLAK peptide from LYZ could not be detected in any of the samples.

#### 2.1.3. Optimization of Protein Elution

From the 18 examined peptides, 9 (DLYNFLK from S100A9, LLGDFFR from LL-37, TFVPGCQPGEFTLGNIK from LCN2, ALNSIIDVYHK and GADVWFK from S100A8, VPLQQNFQDNQFQGK from LCN2, ENAGEDPGLAR from DCD, SILLTEQALAK from LACRT, WESGYNTR from LYZ) could be detected in either healthy or inflamed skin samples as demonstrated by the fact that these peptides showed coelution with their SIL counterparts. The other 9 peptides were not present at a detectable level in the examined samples. In order to elucidate whether the failure to detect 9 peptides was due to the low protein yield obtained with the hexane-based elution the effect of elution on protein recovery was tested. In total, 10 SC samples from the forehead of a healthy volunteer (male, age 30) were collected and discs were divided equally into 2 groups: one group was used for hexane-free and another one for hexane-based elution method [28,38].

**Figure 2:**
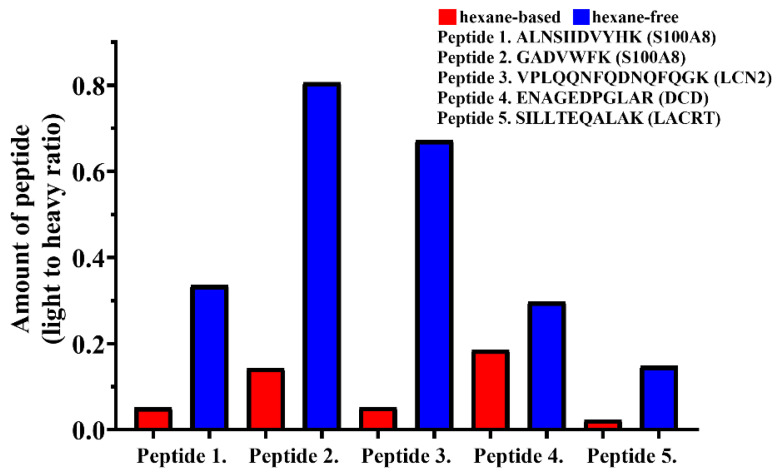
Comparison of the amounts of the selected peptides following a hexane-based or a hexane-free peptide elution. On the ‘y’ axis the number of peptides expressed in form of light to heavy ratios observed in case of examined peptides is plotted. The red bars represent the ratios observed in case of samples obtained by hexane-based elution, while the blue bars show the values observed in case of hexane-free elution.

The proteins eluted from the discs either with the hexane-based or hexane-free method were digested and examined with mass spectrometry. For comparison 5 peptides from 4 proteins previously detected in both sample types (Table 2) were used. The light-to-heavy ratio often used for relative quantification [39] was calculated in case of both elution types (Figure 2). The ratio refers to the peptide amount in the sample normalized to the amount of synthetic SIL peptide; higher ratios indicate higher peptide amounts in the sample.

According to our observations the extracted peptide amounts were markedly higher in hexane-free elution compared to the hexane-based elution in SC samples. Therefore, the hexane-free method was applied for the examination of the AMPs in all further experiments.

### 2.2. Comparative Analysis of AMP Levels of Different Healthy Skin Regions

**Figure 3:**
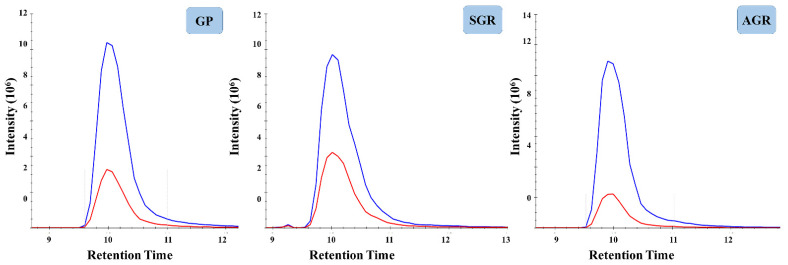
Representative PRM spectra of dermcidin’s ENAGEDPGLAR peptide. The ‘y’ axis shows the intensity of chromatographic peaks while the ‘x’ axis shows the retention time (min). Blue lines refer to the synthetic, SIL peptides, while red lines indicate the endogenous peptides. GP refers to gland poor, SGR represents sebaceous gland rich and AGR the apocrine gland rich healthy skin regions.

Following the optimization experiments, the comparative analyses of skin-derived AMPs in GP, SGR and AGR healthy skin regions were performed. SC samples were collected from GP, SGR and AGR skin regions of 15 healthy volunteers (10 women, 5 men) (Table S1). SC samples were collected from forearm, forehead and armpit, representing GP, SGR, and AGR skin regions, respectively. The collected samples were examined by the optimized sample elution, digestion and targeted proteomics analysis workflow. The light to heavy ratios for peptides of the 18 skin-derived AMPs were determined in the samples collected from GP, SGR and AGR skin regions (Figure 3, Figure S1).

**Figure 4:**
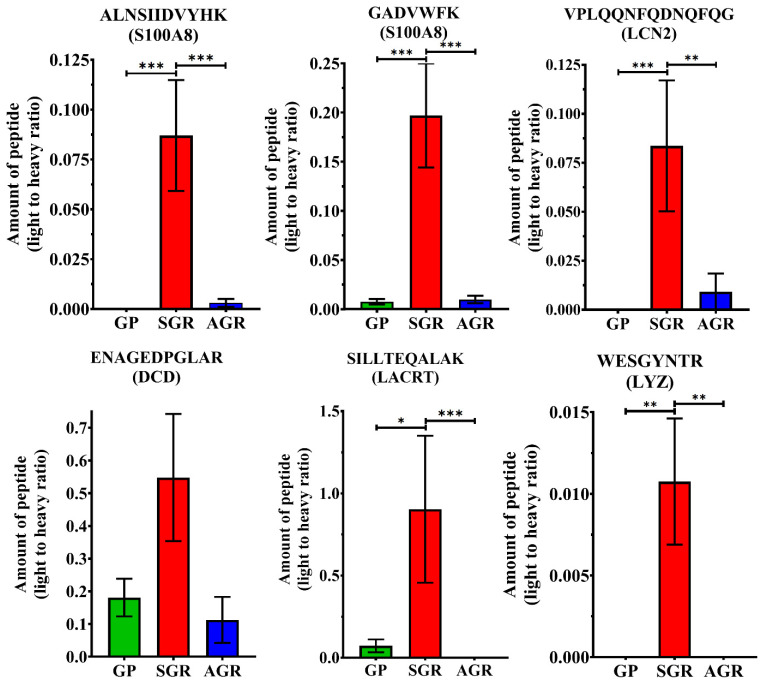
The protein level of examined AMPs in different skin regions. On the ‘y’ axis the mean ± SEM interval of light to heavy ratios were plotted for the skin regions shown on ‘x’ axis. * indicates statistically significant difference (* *p* < 0.05; ** *p* < 0.01; *** *p* < 0.001). GP: sebaceous and apocrine gland poor; SGR: sebaceous gland rich; AGR: apocrine gland rich skin areas.

ALNSIIDVYHK and GADVWFK peptides of S100A8, VPLQQNFQDNQFQGK peptide of LCN2, ENAGEDPGLAR peptide of dermcidin, SILLTEQALAK peptide of LACRT, WESGYNTR peptide of the LYZ could be detected and, as expected, coeluted with their SIL synthetic counterparts and were suitable for quantification (Figure S1). ALNSIIDVYHK peptide of S100A8 and VPLQQNFQDNQFQGK peptide of LCN2 could not be detected, but GADVWFK peptide of S100A8 was detectable in low amounts in GP skin, while in AGR skin, these peptides were present in higher amounts. These peptides could be detected in significantly elevated levels in SGR skin area compared to AGR and GP skin regions. The SILLTEQALAK peptide of LACRT and the WESGYNTR peptide of the LYZ could not be detected in AGR skin, however SILLTEQALAK peptide of LACRT was present in GP skin in low amount, and the WESGYNTR peptide of the LYZ was undetectable. These two peptides were detected in relatively high amounts in SGR skin region and the difference between SGR and GP and SGR and AGR, respectively, was statistically significant. ENAGEDPGLAR peptide from DCD could be quantified in samples originating from all skin regions; however, the amounts did not show any significant topographical differences among the skin regions (Figure 4).

## 3. Discussion

Through their antimicrobial and immunomodulatory effects AMPs are important players of skin homeostasis. By suppressing/preventing the invasion of potential pathogens, some AMPs are considered as markers reflecting the defensive status of the skin [1,7,21,23,40,41]. Additionally, as cohesive forces gradually increase with depth, the AMPs that can be retrieved by a tape stripping approach frequently applied for superficial skin sampling might be of highest relevance [30]. Furthermore, the outermost surface of the skin represents the first line of defence upon contact with microbiota, making the surface and the top layer of the skin the most valuable area for retrieving diagnostic material [27].

In addition, the level of AMPs can have a predictive value and under pathological conditions they may reflect the severity of the inflammation [7]. So far, the most commonly used methods for the molecular analysis of the skin rely on invasive sampling, such as biopsies, and are associated with scarring. There is an increasing demand for novel, less invasive sampling methods. With such a procedure, relevant epidermal sampling could be conducted repeatedly from disease skin regions [23,40] without inflicting pain or discomfort to patients or study subjects.

Proteomes of the SC layers can be examined by minimally invasive sampling using self-adhesive tape strips [8]. Samples collected with tape stripping method provide suitable material for the identification of SC-specific proteins [27]. In this way, hBDs, LL-37, RNase 7, S100A7, DCD proteins have been identified in the SC samples of healthy and inflamed skin [1,2,14,20,22,24,25].

Our previous studies at the mRNA and the protein levels demonstrated significant differences in the expression levels of the S100A7, S100A8, S100A9, hBD2, LCN2 and TSLP among the different healthy skin regions [2,24,25]. Beside them, in our current study we investigated further AMPs (hBD1, 3, 4, LL-37, LACRT, LYZ and DCD), which are known to be present in the epidermis under both steady-state and diseased conditions. [7,42,43,44].

In this study, we developed a novel, rapid and minimally invasive tape stripping approach for the sample collection and characterization of secreted AMPs of the SC of normal skin. In our study, the outermost layer of the SC was removed using D-Squame disks. While, earlier several groups reported the retrieval of the protein content from the adhesive tape by using a hexane-based solvent [28,38], in our study the proteins captured on the surface of these disks were eluted using a novel, hexane-free extraction procedure. In comparison, the biomaterial collected by the hexane-free approach exceeded the amounts obtained by hexane containing solvents. In the case of the hexane-free elution, proteins were denatured from the surface of tape directly. In contrast, during hexane-based method the disks carrying the proteins were glued onto silane-covered slides and then washed in hexane before denaturation. The step preceding denaturation may have resulted in protein loss, but this hypothesis requires further testing.

Previously, proteins obtained by the tape stripping method were analyzed by ELISA, qPCR or immunohistochemistry [21,26,27,28]. On the contrary, our method is based on a highly specific and sensitive PRM-based mass spectrometry detection [29].

Several studies reported the use of six or more discs to obtain specimen from the same place of the skin surface, discarding the first tape of the series [23,30,40]. Considering the discomfort provoked by the consecutive stripping from the same place, we omitted repetitive samplings and, in our optimized method, only one disc was collected from one site.

In our previous investigations using qPCR and imaging methods on skin biopsies, we demonstrated that significant topographical immunological differences exist among AMPs from various regions of the epidermis under steady-state (among GP, AGR, and SGR regions) as well as in inflammatory skin conditions [2,24,25].

In the current study, our previous findings were recapitulated based on SC specimens obtained by tape stripping technique and quantification of AMPs by PRM-based mass spectrometry. ALNSIIDVYHK and GADVWFK peptides of S100A8, VPLQQNFQDNQFQGK peptide of LCN2, ENAGEDPGLAR peptide of dermcidin, SILLTEQALAK peptide of LACRT and WESGYNTR peptide of the LYZ could be detected in different healthy skin regions.

Peptides originated from S100A8, LCN2, DCD, LACRT and LYZ proteins, each characterized by a functional role in antimicrobial defense of the skin, were identified and quantified [3,7,42,45]. S100A8, LCN2, LACRT and LYZ proteins were detected in significantly higher levels in SGR regions compared to GP and AGR skin regions. We also found that ALNSIIDVYHK and GADVWFK peptides of S100A8 and VPLQQNFQDNQFQGK peptide of LCN2 were present in notably higher amounts in AGR skin compared to GP skin and could be detected in significantly increased amounts in SGR skin compared to GP and AGR skin regions. In accordance with our previous findings, these data suggest that healthy SGR and AGR skin regions have a more pronounced immune tuning relative to GP area [2,25]. Moreover, we could identify two previously unexamined AMP molecules (SILLTEQALAK peptide from LACRT and WESGYNTR peptide from LYZ), which were detected in significantly higher amounts in SGR skin compared to GP and AGR skin areas. The absence of LCN2, LYZ and the low level of S100A8 and LACRT suggest that GP skin might be associated with the less pronounced steady-state immune activity [2,24,25].

The amount of DCD produced by eccrine glands was detected to be comparable in the three healthy regions, which is consistent with previous findings [20]. Since DCD is present in the sweat of the eccrine gland in healthy skin, but its amount is significantly reduced or completely disappears during inflammation, its altered level can indicate inflammatory skin diseases in their initiation phase [20,22,42].

AMPs play an important role in the development and pathogenesis of certain skin diseases and have significant impact on their severity. Therefore, detection of AMPs using a minimally invasive tape stripping technique can be useful both in scientific investigation as well as in clinical follow-up of skin diseases such as AD, HS, PPR or Ps. With this painless technique, the number of AMPs can be detected quickly, therefore, it can be a suitable method for gaining information regarding the severity of these skin diseases and investigating the efficacy of therapies [23].

## 4. Materials and Methods

### 4.1. Subjects

All volunteers involved in this study were recruited from the outpatient clinic of the Department of Dermatology, Faculty of Medicine, University of Debrecen and written informed consent was obtained prior to SC sample collection. Sample collection was performed according to the guidelines of Helsinki Declaration and this study was approved by the local ethics committee of the University of Debrecen, Hungary (ethical approval nr. 5064—2018—DE RKEB/IKEB, 17 September 2018). Samples from patients suffering from inflammatory skin diseases (atopic dermatitis [AD], hidradenitis suppurativa [HS], psoriasis vulgaris [Ps], papulopustular rosacea [PPR] with moderate to severe skin symptoms) were collected before starting any treatment or medication. Healthy volunteers did not have a history or any manifestations of inflammatory skin diseases. The applied sample collection method was simple, fast, minimally invasive and completely painless and harmless for the participants.

A total of 60 samples from 23 adult volunteers (19 healthy controls and 4 patients, Table S1) were collected and divided into optimization and test groups. The optimization group contained three subgroups used for (1) optimization of sample collection (samples from 2 healthy volunteers: 1 female, age: 41 years; 1 male, age: 34 years), (2) design and optimization of PRM-based mass spectrometry method (samples from 1 female patient with severe HS, age: 44 years; 1 male patient with moderate Ps, age: 30 years; 1 female patient with severe PPR, age: 40 years; 1 male patient with moderate AD, age: 44 years; 1 healthy male donor, age: 34 years) and (3) optimization of protein elution (samples from forehead region of 1 healthy male donor, age: 34 years), according to the study workflow (Figure S2). The test group included samples from 15 healthy volunteers: 10 female, median age: 28 ± 9.7 years; and 5 male donors, median age: 35 ± 10.8 years.

Only healthy donors, without any sign of skin or systemic inflammation have been recruited into the study, while patients suffering from different immune-mediated skin diseases had moderate to severe symptoms and did not receive systemic or local medications prior to sampling.

### 4.2. Sample Collection

SC samples were collected from the skin surface with tape stripping technique using D-Squame discs (Clinical and Derm LLC, Walsall, West Midlands, UK). In the course of each examination, D-Squame discs were always used by the same person wearing powder-free latex gloves. All volunteers were asked not to wash the area of sampling with soap, not to use any cosmetics 24 h prior to sample collection. No disinfectant was used to clean the skin surface before sampling. D-Squame discs were pressed to the skin surface for 10 s with medium force before being peeled off. All D-Squame discs were stored in a wet chamber with samples facing upwards at room temperature until processing. Sample processing was carried out on the sampling day.

### 4.3. Measurement of Protein Concentration

Discs containing SC samples were placed in 10 mL of 0.1% SDS and were sonicated for 2 min. Then, samples were centrifuged for 10 min at 4 °C, and 5500 rpm. Next, samples were dried and re-dissolved in 2 mL ultra-pure water. Protein concentration was determined using the Pierce BCA Protein Assay Kit (ThermoFisher Scientific, Waltham, MA, USA) according to the manufacturer’s instruction in 96 well plastic plates. A 0.1% SDS was used as negative control. The protein measurement was performed at 595 nm wavelength on a Labsystems Multiskan MS spectrophotometer (ThermoFisher Scientific, Waltham, MA, USA).

### 4.4. Protein Elution from D-Squame Discs

Proteins from the collected tape stripping discs have been eluted with hexane as previously described [28,38]. Briefly, the D-Squame discs removed from the skin surface were glued to silane-coated slides (Sigma-Aldrich Ltd., St. Louis, MO, USA) and soaked in hexane (Sigma-Aldrich Ltd., St. Louis, MO, USA) at room temperature, overnight. The proteins were denatured on the discs with 8 M urea (Sigma-Aldrich Ltd., St. Louis, MO, USA) for 30 min at room temperature in a wet chamber by evenly spreading urea solution across the disc surface. The solution of denatured proteins was collected to Eppendorf tubes for digestion.

To improve the recovery of skin-derived AMPs from the SC, a hexane-free elution method was applied. The hexane-based elution method was modified as follows: after removal from the skin the D-Squame discs were placed in a wet chamber with samples facing upwards and 8 M urea solution was added immediately and the denatured proteins were collected to Eppendorf tubes.

### 4.5. Protein Digestion

The proteins eluted from the discs were digested with trypsin. Reduction was carried out using 10 mM dithiothreitol (Bio-Rad, Hercules, CA, USA) for 1 h at 37 °C, followed by alkylation with 20 mM iodoacetamide (Bio-Rad, Hercules, CA, USA) for 45 min at room temperature in the dark. Before digestion, urea was diluted to 1 M with 25 mM ammonium bicarbonate (Sigma-Aldrich Ltd., St. Louis, MO, USA). Finally, trypsin digestion was carried out overnight at 37 °C using stabilized MS grade TPCK-treated bovine trypsin (Sigma-Aldrich Ltd., St. Louis, MO, USA) applying a 1:25 trypsin: protein ratio. The digested peptides were dried in speed-vac (ThermoFisher Scientific, Waltham, MA, USA) and then, re-dissolved in 100 µL 1% formic acid and desalted with C18 Pierce Tip (ThermoFisher Scientific, Waltham, MA, USA). The eluates were dried and kept at −20 °C until mass spectrometry analysis. Prior to the injection to the mass spectrometer the peptides were re-dissolved in 10 µL of 1% formic acid (VWR International, LLC, Radnor, PA, USA).

### 4.6. PRM Assay Design

The amino acid sequences of the 13 selected AMPs (calcium-binding protein A8 (S100A8), calcium-binding protein A9 (S100A9), cathelicidin (LL-37), dermcidin (DCD), human beta defensin (hBD) 1, hBD2, hBD3, hBD4, lacritin (LACRT), lipocalin (LCN) 2, lysozyme (LYZ), psoriasin (S100A7), thymic stromal lymphopoietin (TSLP) short isoform) characteristic for the skin surface were retrieved from UniProt database (www.uniprot.org) [46]. In silico trypsin digestion of the protein sequences were carried out using the ExPaSy PeptideCutter (https://web.expasy.org/peptide_cutter/) [47] tool accessible from the UniProt. In order to collect unique, protein-specific sequences, the peptides with 100% cleavage probability and 5–14 amino acid length were subjected to BLASTp search (https://blast.ncbi.nlm.nih.gov) [48] using default settings, the NCBI non-redundant database and “Homo sapiens” as the query species. The protein-specific unique peptide sequences were used for PRM analyses and their stable isotope labeled (SIL) counterparts were ordered from JPT Peptide Technologies GmbH, Berlin, Germany.

### 4.7. Mass Spectrometry Experiment

Digested samples were randomized and analyzed in duplicates under identical conditions. Before the mass spectrometry analyses the same amount of SIL synthetic peptide mixture was added to each sample.

PRM analyses were carried out on an Easy nLC1200 nanoUPLC (ThermoFisher Scientific, Waltham, MA, USA) coupled to Orbitrap Fusion mass spectrometer (ThermoFisher Scientific, Waltham, MA, USA).

Peptides were separated using a 60-min water-acetonitrile gradient at a flow rate of 300 nL/min on an Acclaim PepMap RSLC (150 mm × 50 μm, 2 μm particle size, 100 Å pore diameter, ThermoFisher Scientific, Waltham, MA, USA) column after desalting on Aclaim PepMap C18 (20 mm × 75 μm, 3 μm particle size, 100 Å pore diameter, ThermoFisher Scientific, Waltham, MA, USA) column. The buffer A was LC grade water with 0.1% formic acid (VWR International, LLC, Radnor, PA, USA) and buffer B was LC grade acetonitrile with 0.1% formic acid (VWR International, LLC, Radnor, PA, USA). During the chromatography, buffer B ratio was increased from 5% to 20% over 5 min followed by an increase to 45% over 40 min. Next, the buffer B ratio was increased to 85% over a 3 min period and kept at 85% for 5 min. Then, buffer B ratio was decreased to 5% in 1 min, and held constant for 6 min.

PRM analyses were performed with targeted MS2 approach. The peptide masses were set as precursor ions. After precursor ion selection in the quadrupole analyzer the peptides were fragmented with 30% normalized CID energy, then, fragments were analyzed in the 114–1000 *m*/*z* range in the Orbitrap analyzer (50,000 resolutions, AGC target: 1.0 × 10^−4^, centroid mode) in positive polarity mode.

### 4.8. Data Evaluation and Statistical Analysis

For protein quantification, the Skyline software was used [49]. Shapiro-Wilk test was used for normality testing. As far as the data did not follow normal distribution the three groups were compared by Kruskal-Wallis test followed by Dunn’s post hoc analysis. Statistical analyses were carried out by using GraphPad Prism 8.0.1. (GraphPad Software, San Diego, CA, USA). The data were presented as mean ± SEM. The results were considered to be statistically significant when the *p*-value was less than 0.05 (* *p* < 0.05; ** *p* < 0.01; *** *p* < 0.001).

## 5. Conclusions

We developed and optimized a minimally invasive sampling procedure coupled by PRM-based mass spectrometry method for the examination of AMPs located in the SC of healthy and diseased skin. This novel approach is suitable for the comparison of AMP content of different skin regions as well as healthy and diseased skin samples. Our data suggest that investigation of SC by this minimally invasive method is suitable for the fast and simple examination of AMPs. Understanding the AMP composition of healthy skin surface under steady-state may open new doors for the development of novel therapeutics, moreover, it provides a notable diagnostic and predictive value in the clinical practice, especially for disease follow up and monitoring the efficacy of therapies.

## Figures and Tables

**Table 1:** List of protein-specific unique peptides characteristic to the examined proteins. The gene name, protein name, UniProt ID, peptide sequence and precursor ion (*m*/*z*) of each peptide selected for PRM assay are shown. ‘*’ represents the stable isotope-labelled (SIL) amino acids. Underlined C represents carbamidomethylated cysteines.

Gene Name	Protein Name	UniProt ID	Protein-Specific Unique Peptide	Precursor Ion (*m*/*z*)
S100A8	calcium-binding protein A8	P05109	ALNSIIDVYHK	424.903
			ALNSIIDVYHK *	427.574
S100A8	calcium-binding protein A8	P05109	GADVWFK	411.71
			GADVWFK *	415.718
S100A9	calcium-binding protein A9	P06702	DLYNFLK	456.744
			DLYNFLK *	460.752
LL-37	cathelicidin	P49913	LLGDFFR	434.239
			LLGDFFR *	439.243
DCD	dermcidin	P81605	ENAGEDPGLAR	564.767
			ENAGEDPGLAR *	569.771
hBD1	human beta-defensin 1	P60022	IQGTCYR	449.216
			IQGTCYR *	454.22
hBD2	human beta-defensin 2	O15263	GIGDPVTCLK	530.278
			GIGDPVTCLK *	534.285
hBD3	human beta-defensin 3	P81534	GIINTLQK	443.771
			GIINTLQK *	447.778
hBD4	human beta-defensin 4	Q8WTQ1	ICGYGTAR	449.216
			ICGYGTAR *	454.22
LACRT	lacritin	Q9GZZ8	QELNPLK	421.242
			QELNPLK *	425.249
LACRT	lacritin	Q9GZZ8	SILLTEQALAK	593.855
			SILLTEQALAK *	597.862
LCN2	lipocalin-2	P80188	VPLQQNFQDNQFQGK	895.944
			VPLQQNFQDNQFQGK *	899.951
LCN2	lipocalin-2	P80188	SYNVTSVLFR	593.316
			SYNVTSVLFR *	598.32
LCN2	lipocalin-2	P80188	TFVPGCQPGEFTLGNIK	932.966
			TFVPGCQPGEFTLGNIK *	936.973
LYZ	lysozyme	P61626	GISLANWMCLAK	682.346
			GISLANWMCLAK *	686.353
LYZ	lysozyme	P61626	WESGYNTR	506.727
			WESGYNTR *	511.731
S100A7	psoriasin	P31151	SIIGMIDMFHK	646.33
			SIIGMIDMFHK *	650.337
TSLP	thymic stromal lymphopoietin	Q969D9	CLEQVSQLQGLWR	808.914
			CLEQVSQLQGLWR *	813.918

**Table 2:** List of the identified AMPs in the SC of healthy and inflamed skin samples. AMPs in healthy and inflamed skin were identified by PRM-based mass spectrometry analysis. The examined protein, the characteristic unique peptide along with their presence or absence in the examined sample is listed. ‘+’ sign indicates the presence, while ‘−’ sign indicates the absence of proteins. The proteins are labelled by their gene name.

Protein	Peptide	Healthy Skin	Inflamed Skin
S100A9	DLYNFLK	−	+
LL-37	LLGDFFR	−	+
LCN2	TFVPGCQPGEFTLGNIK	−	+
S100A8	ALNSIIDVYHK	+	+
S100A8	GADVWFK	+	+
LCN2	VPLQQNFQDNQFQGK	+	+
DCD	ENAGEDPGLAR	+	+
LACRT	SILLTEQALAK	+	+
LYZ	WESGYNTR	+	+
S100A7	SIIGMIDMFHK	−	−
hBD1	IQGTCYR	−	−
hBD2	GIGDPVTCLK	−	−
hBD3	GIINTLQK	−	−
hBD4	ICGYGTAR	−	−
LCN2	SYNVTSVLFR	−	−
TSLP	CLEQVSQLQGLWR	−	−
LACRT	QELNPLK	−	−
LYZ	GISLANWMCLAK	−	−

## Data Availability

The data presented are available on https://panoramaweb.org/University%20of%20Debrecen/Skin%20AMP/project-begin.view (accessed on 15 March 2021) website.

## Supplementary Materials

The following are available online at https://www.mdpi.com/article/10.3390/ijms22084233/s1, Table S1: List of the recruited donors, Figure S1: Representative PRM spectra of the examined peptides. ALNSIIDVYHK and GADVWFK peptides of S100A8, VPLQQNFQDNQFQGK peptide of LCN2, ENAGEDPGLAR peptide of dermcidin, SILLTEQALAK peptide of LACRT, WESGYNTR peptide of the LYZ (red curves) show, coelution with their SIL synthetic counterparts (blue curves) indicating the presence of these peptides in the examined samples. Figure S2: Study workflow.
