# Supplementary figures and images for: Detection of Antimicrobial Peptides in Stratum Corneum by Mass Spectrometry
Source: Int J Mol Sci. 2021 Apr 19;22(8):4233. doi: 10.3390/ijms22084233 (PMC8073585; doi:10.3390/ijms22084233)

ALNSIIDVYHK (S100A8)

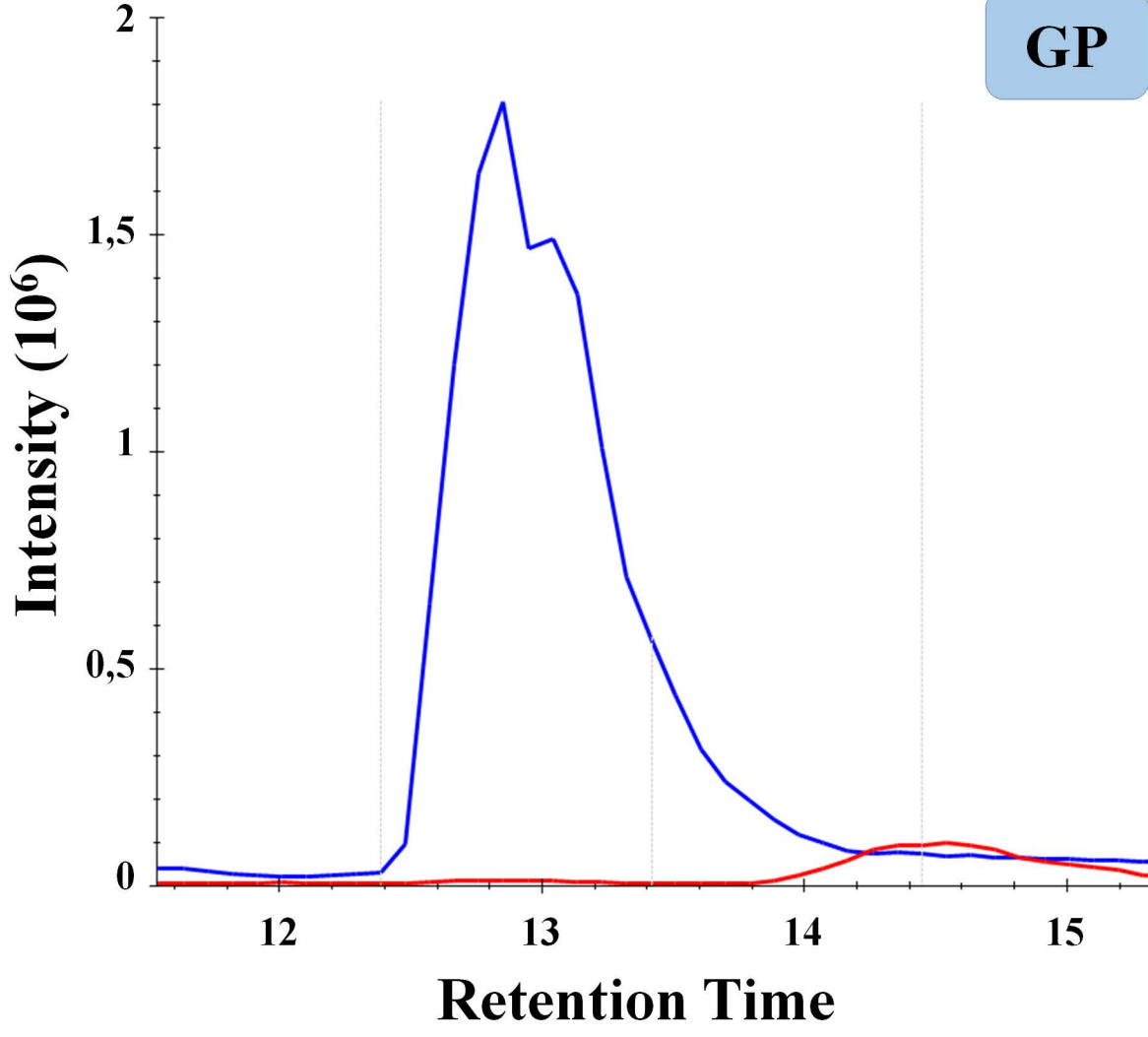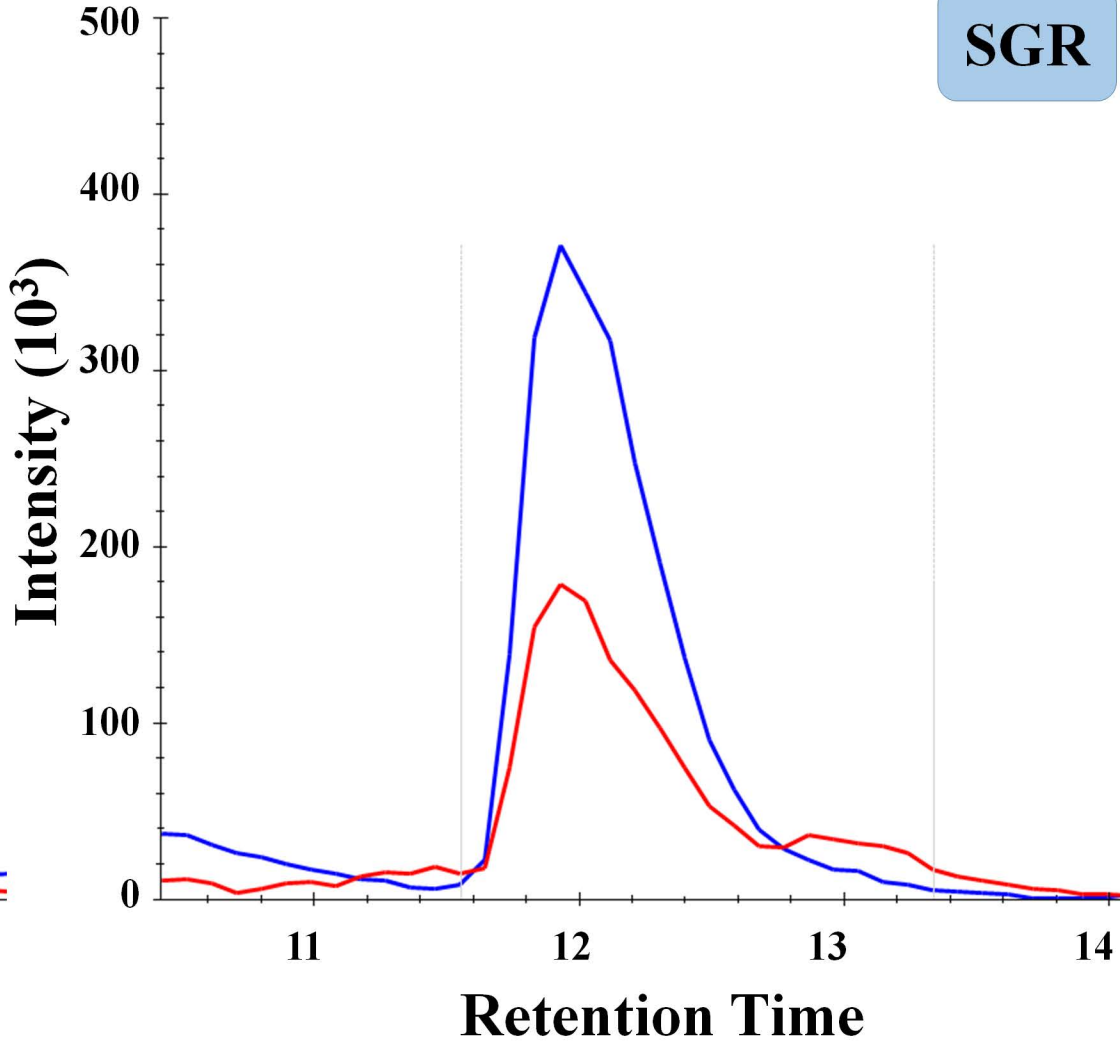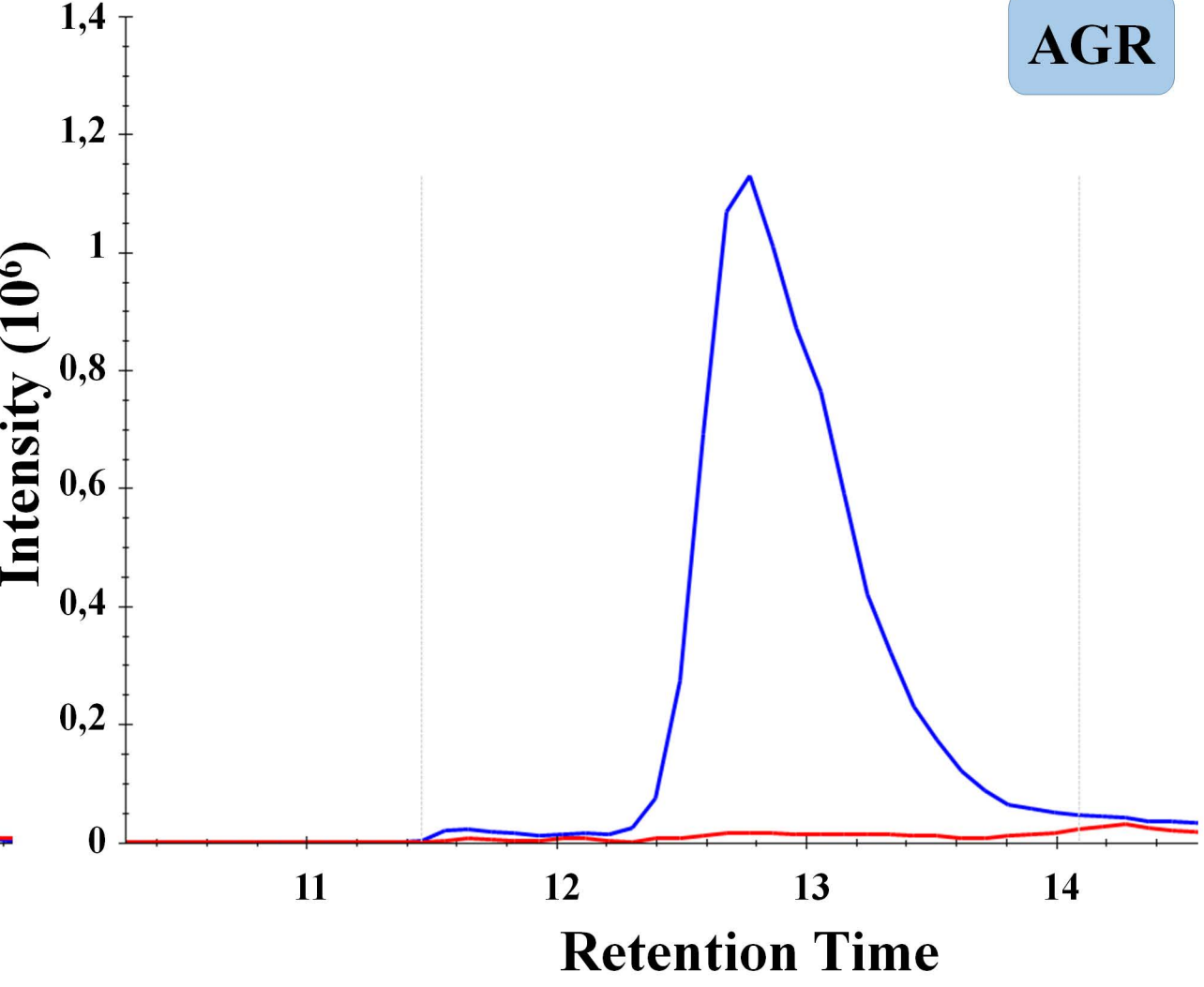

GADVWFK (S100A8)

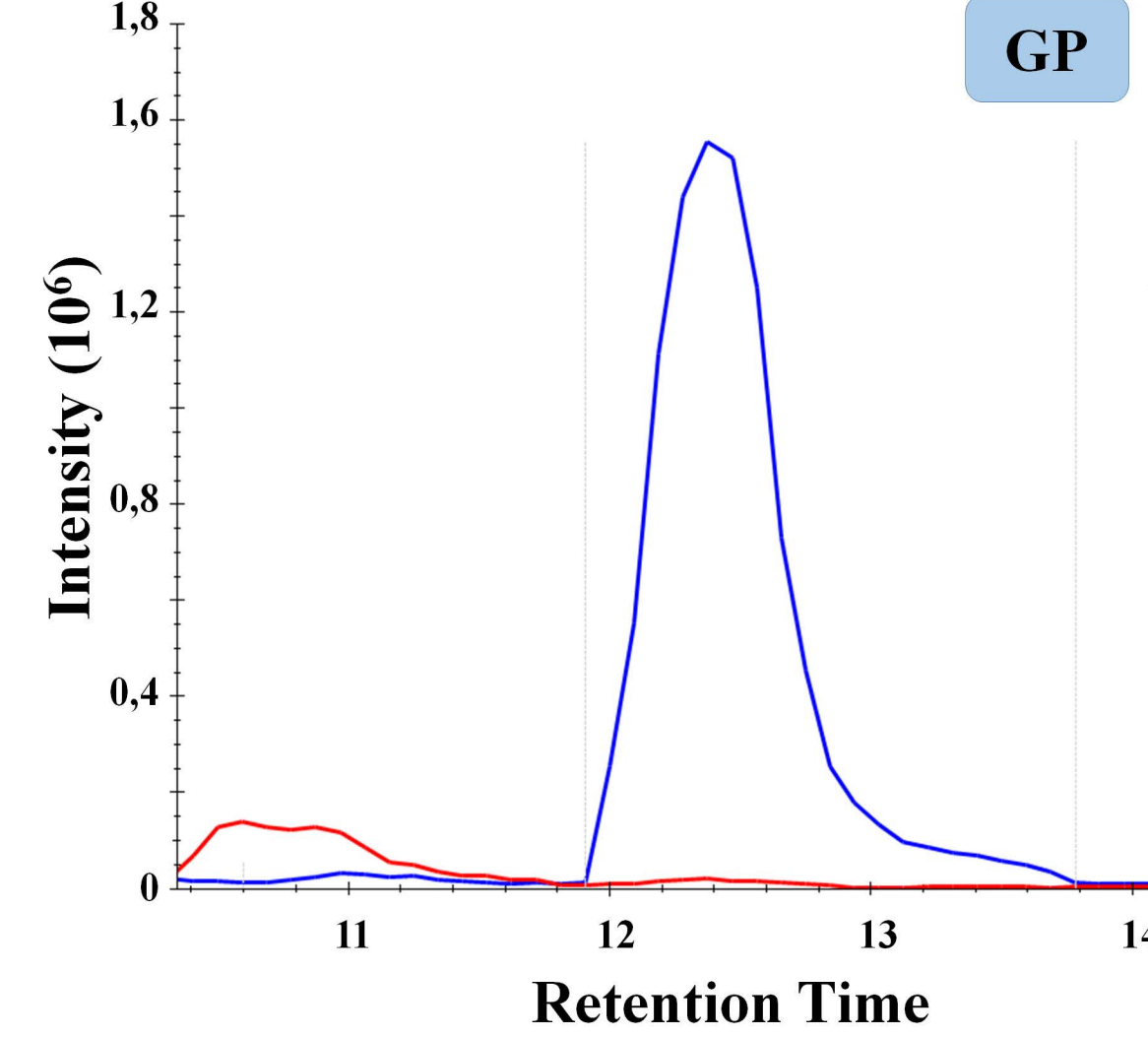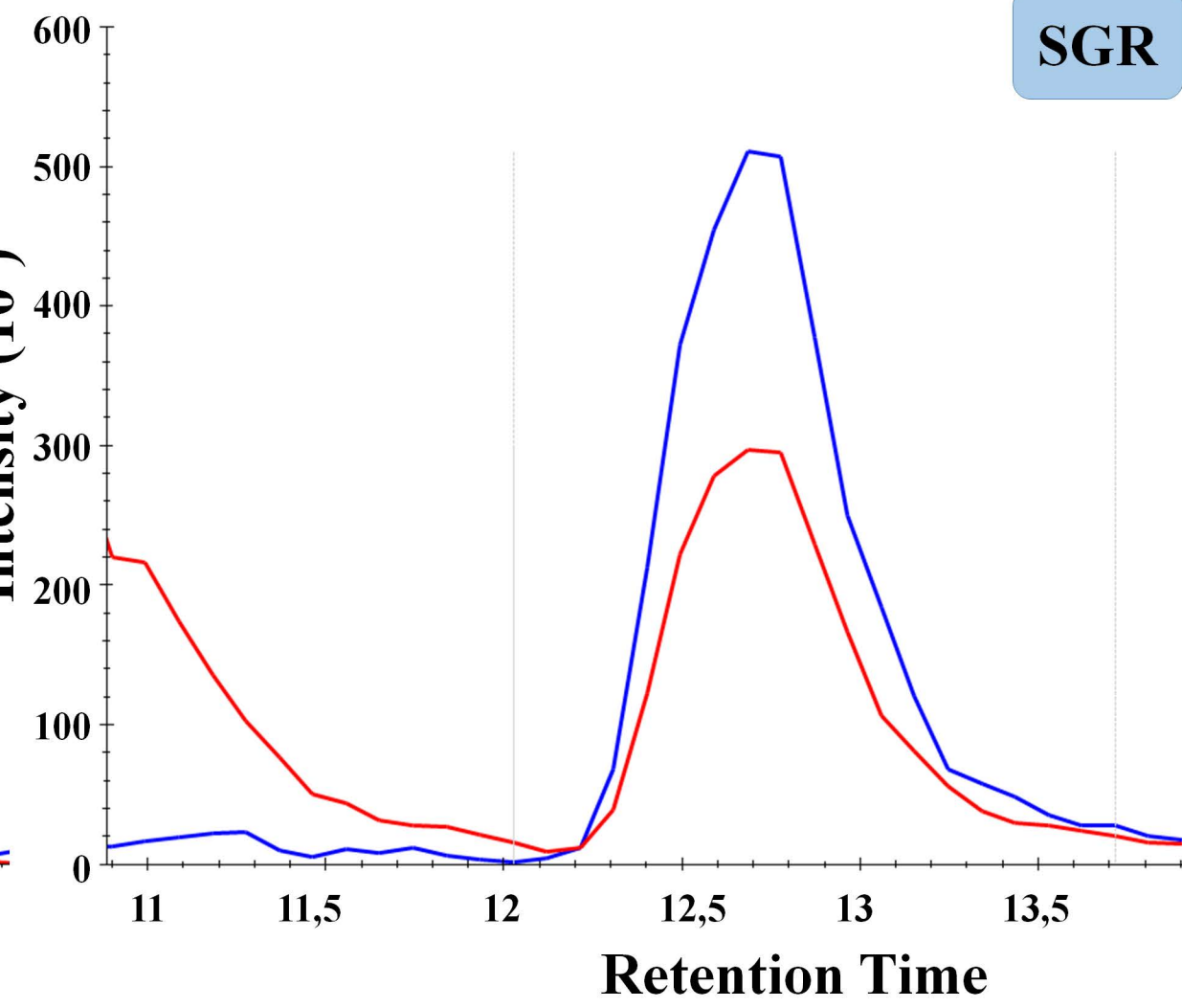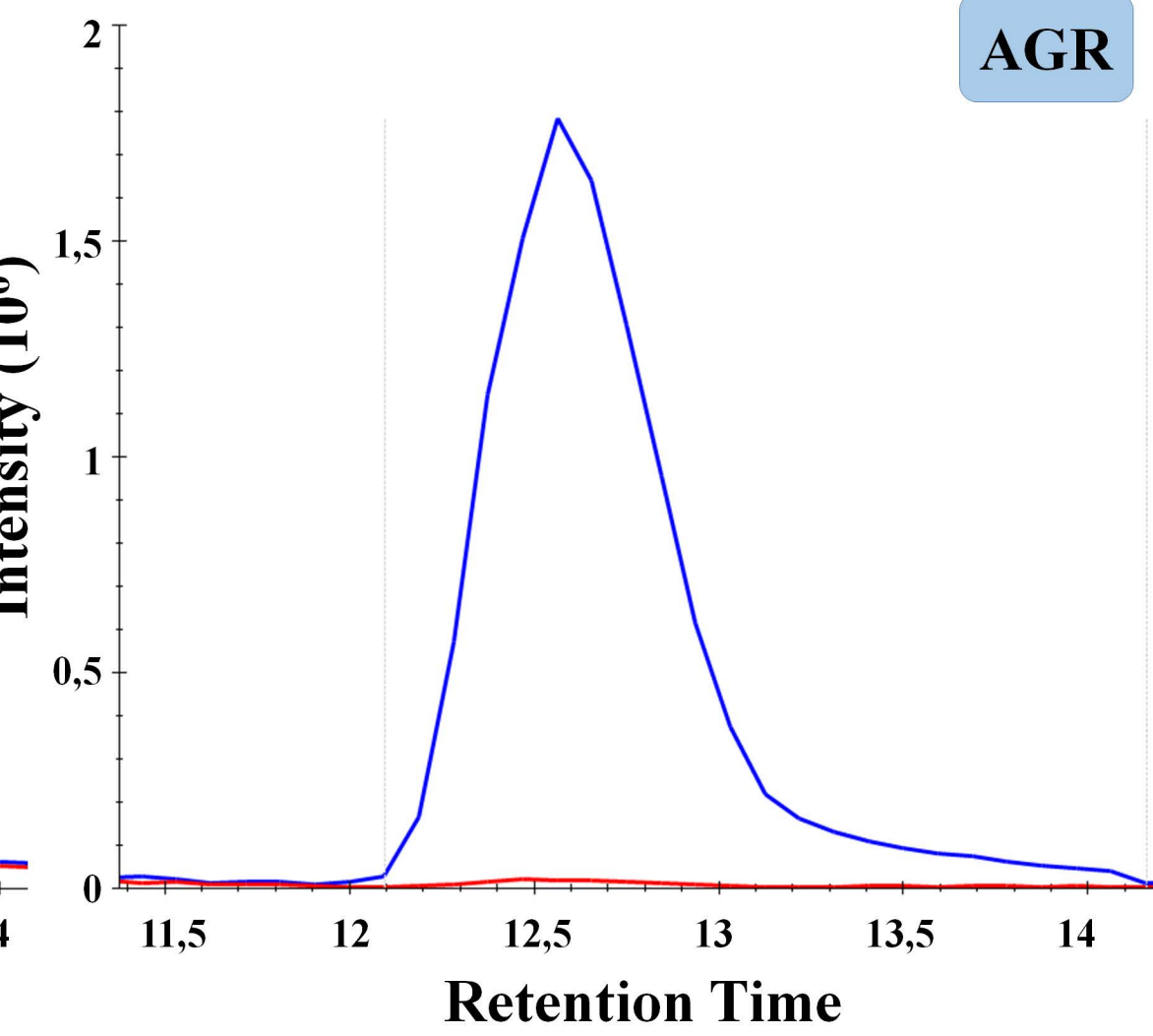

VPLQQNFQDNQFQGK (LCN2)

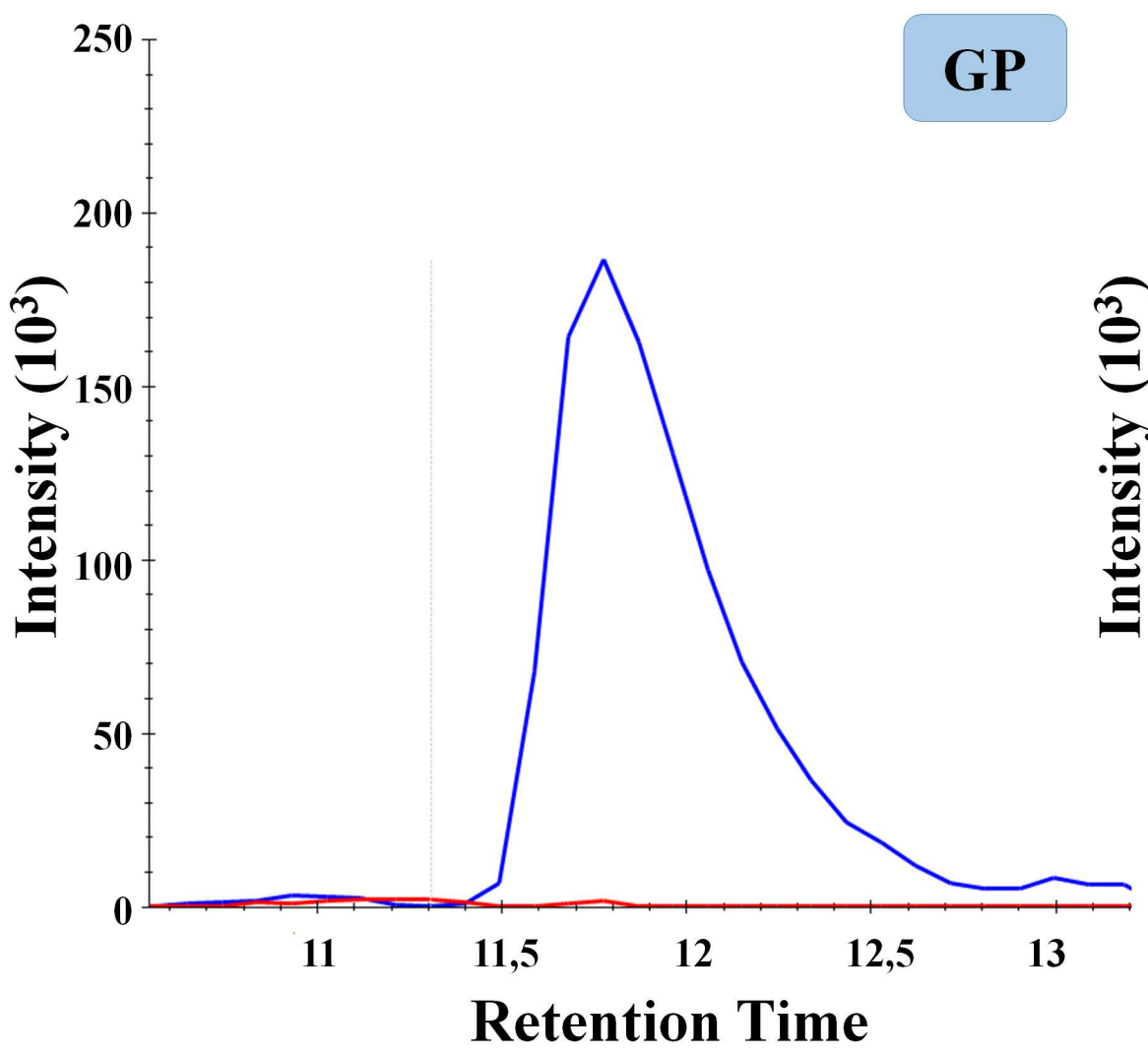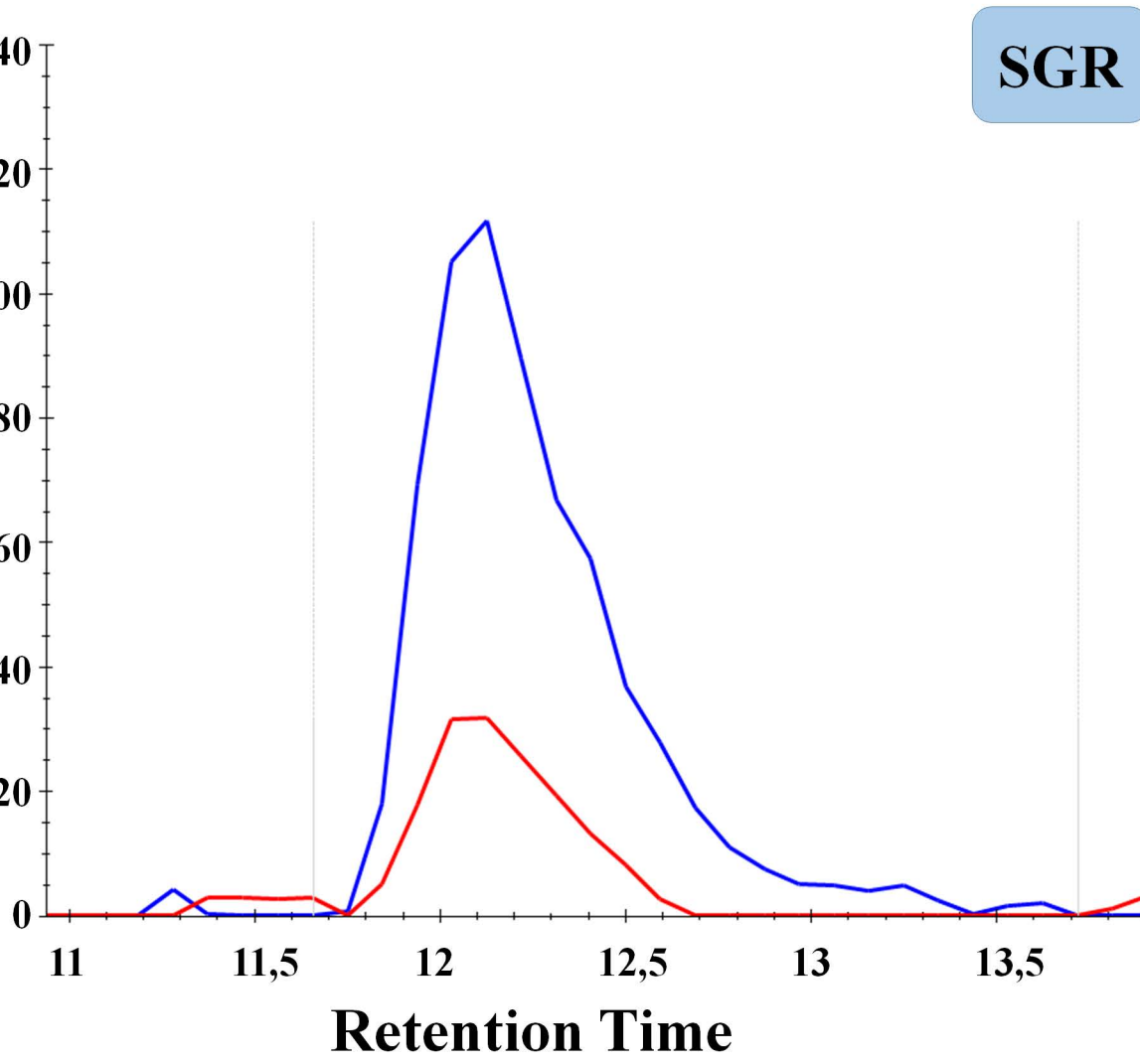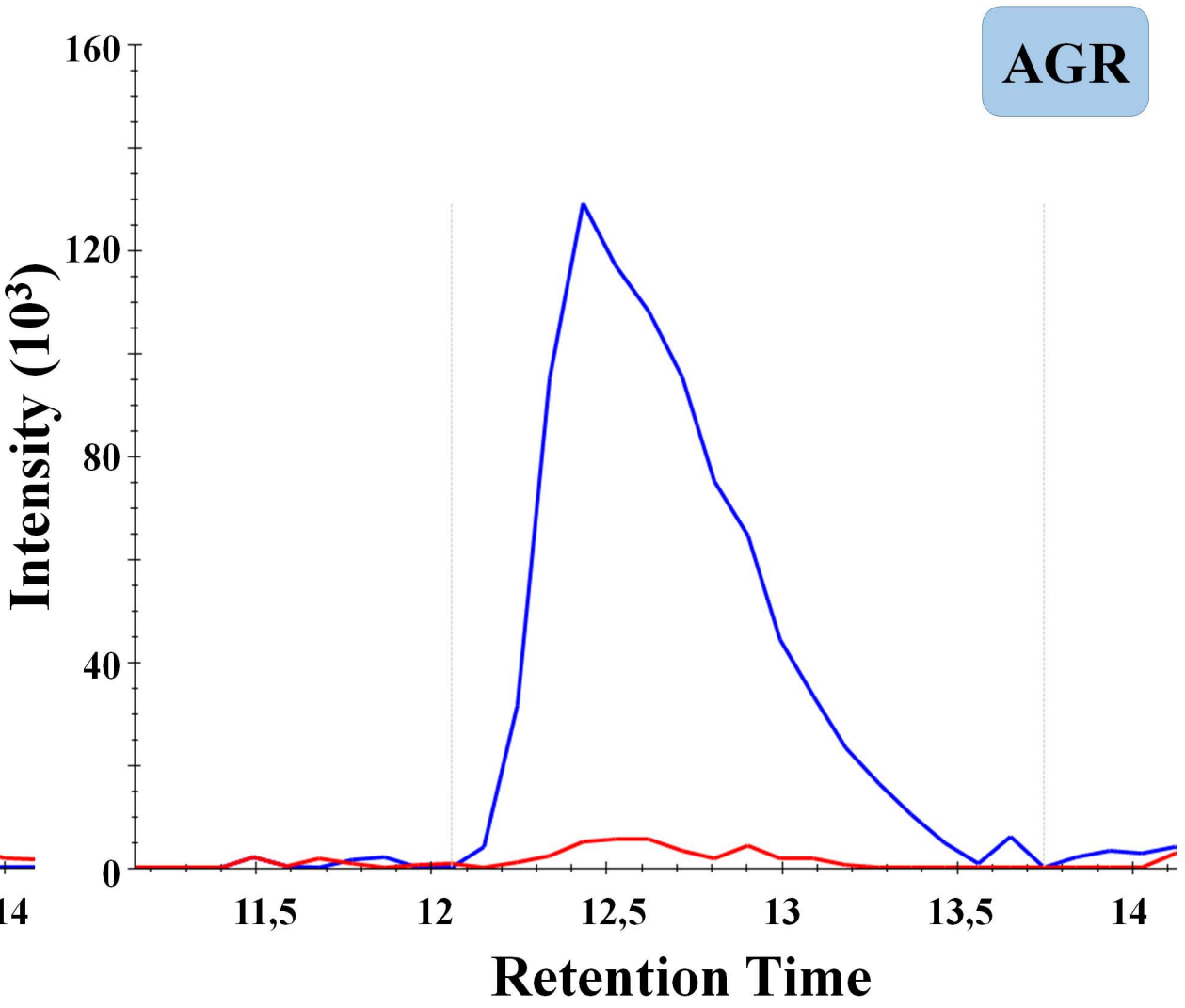

SILLTEQALAK (LACRT)

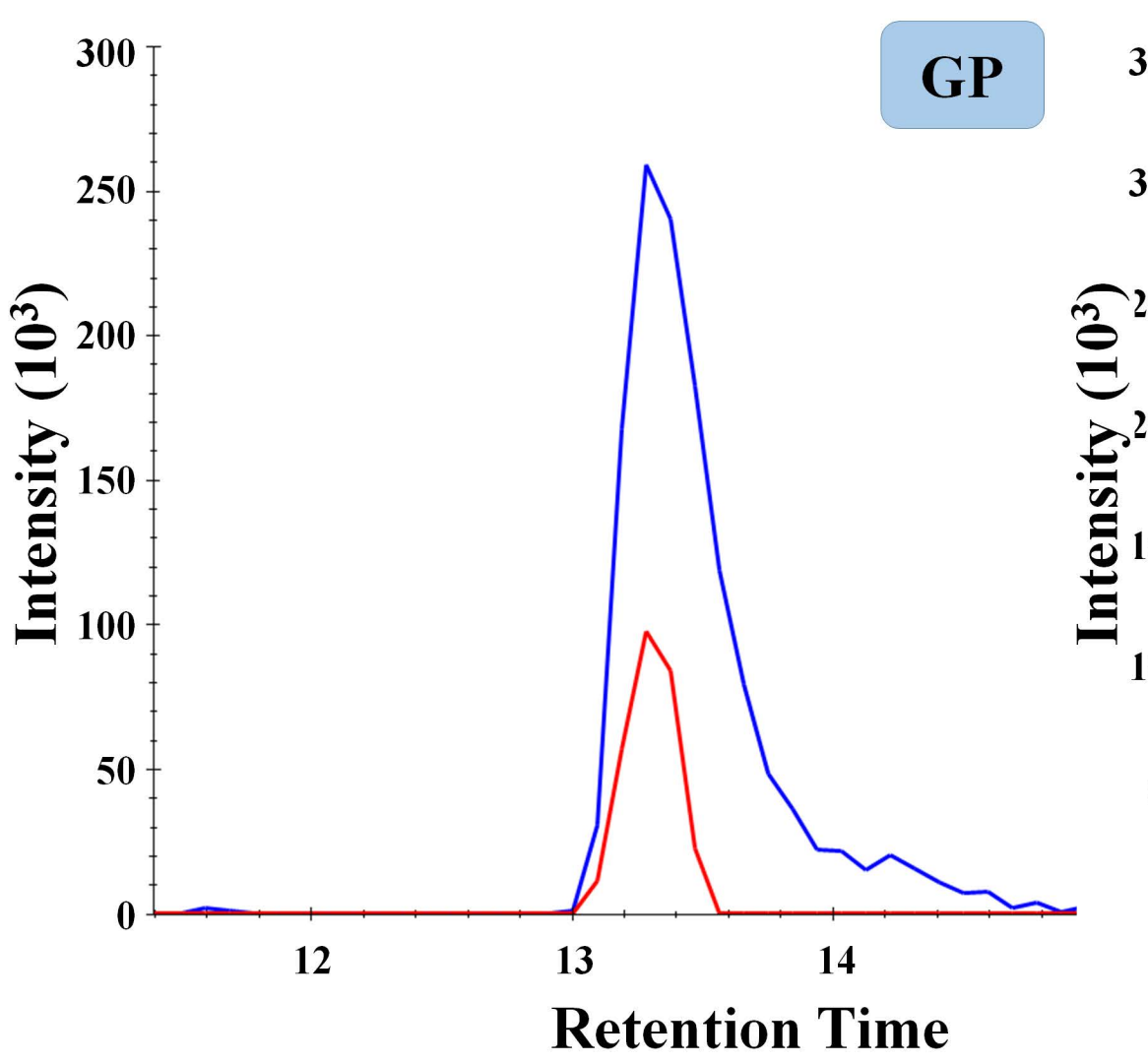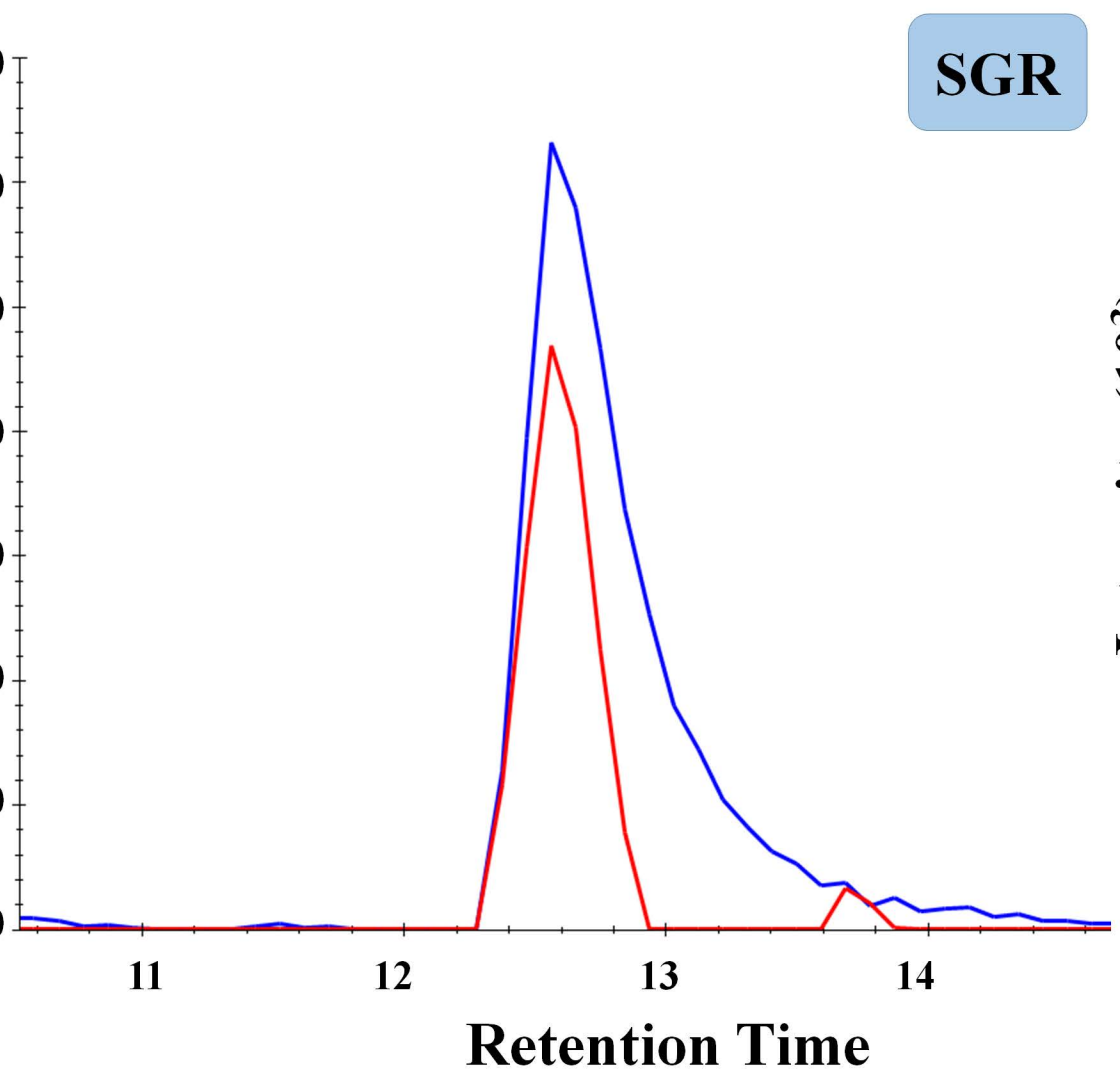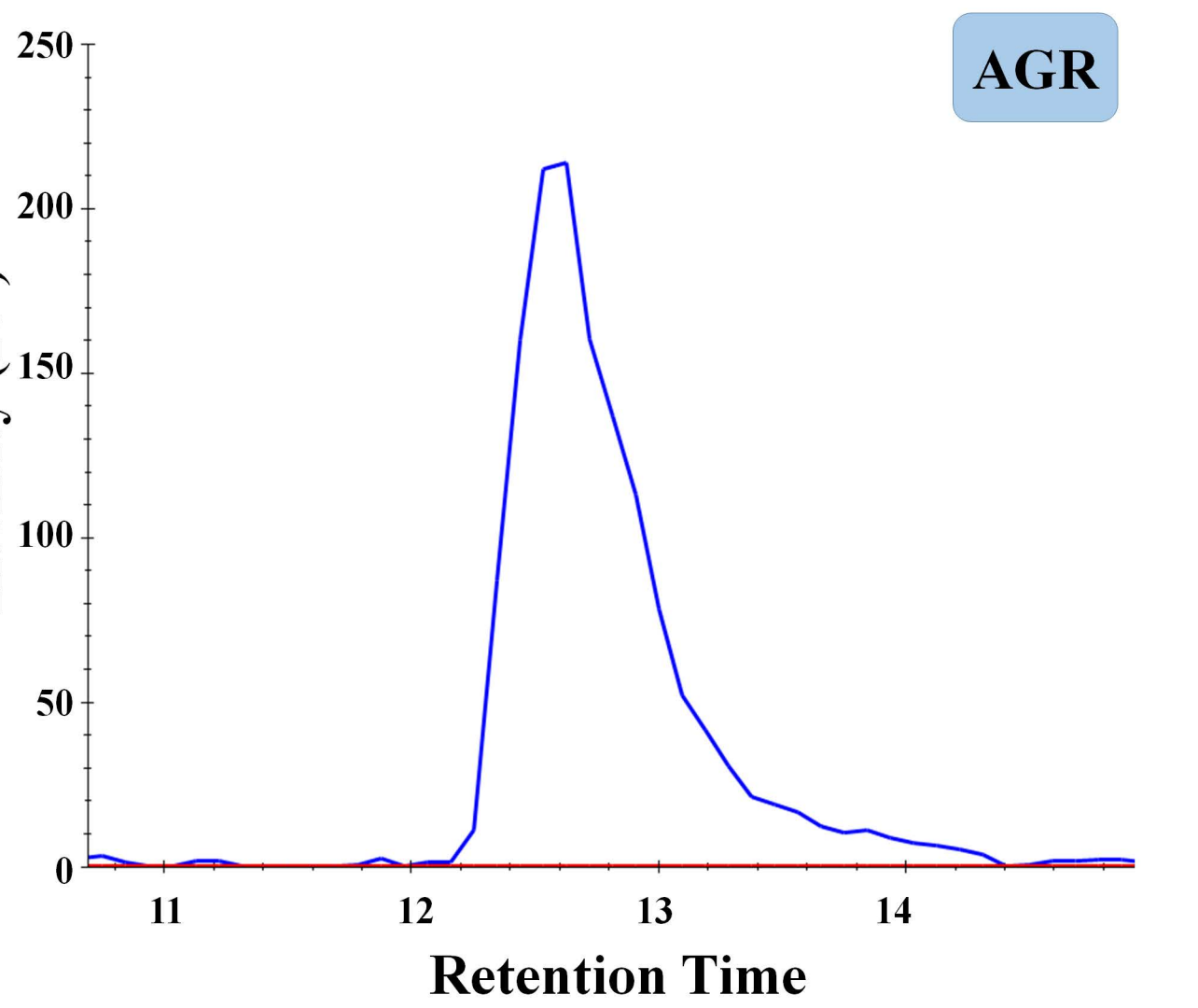

WESGYNTR (LYZ)

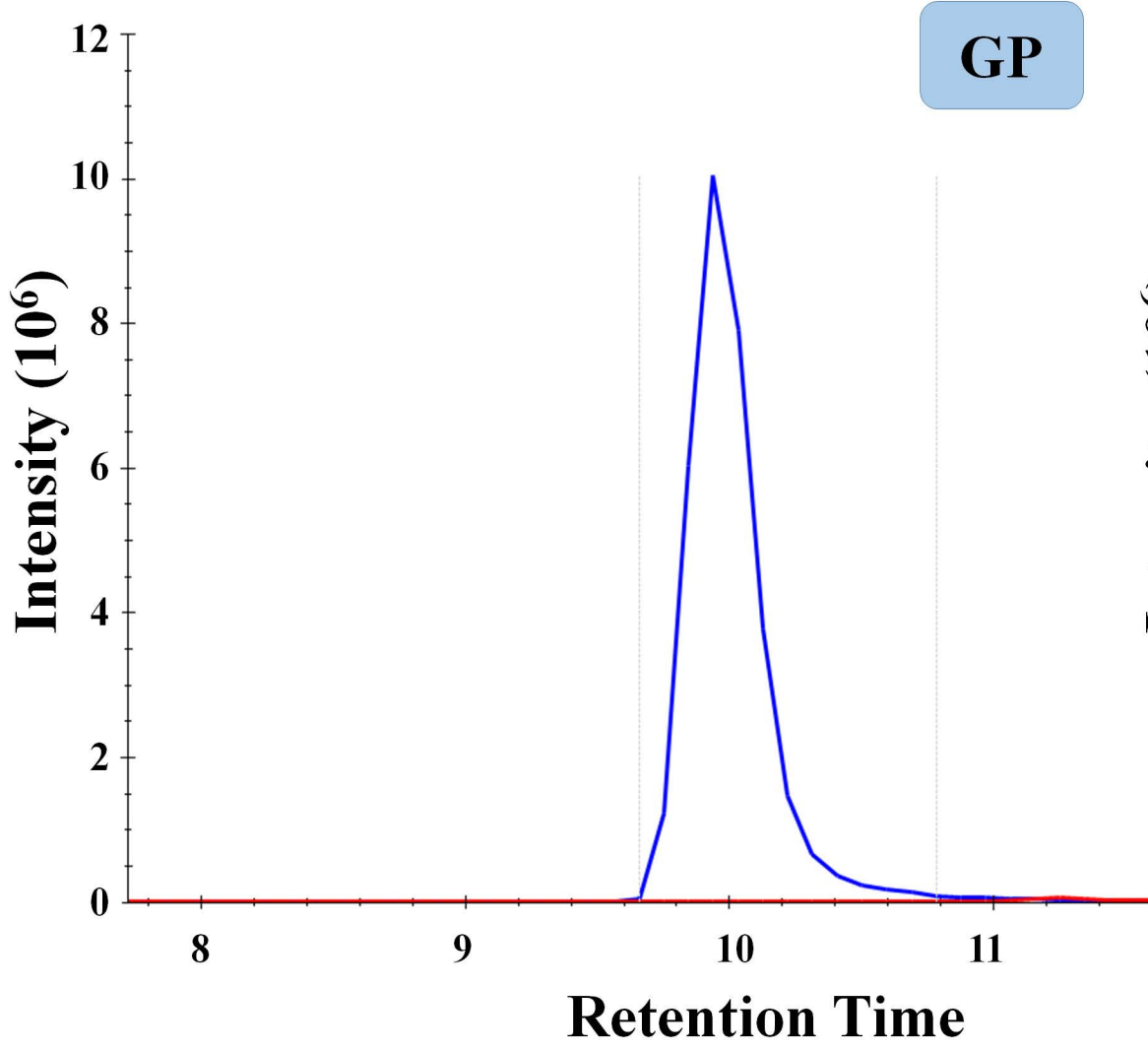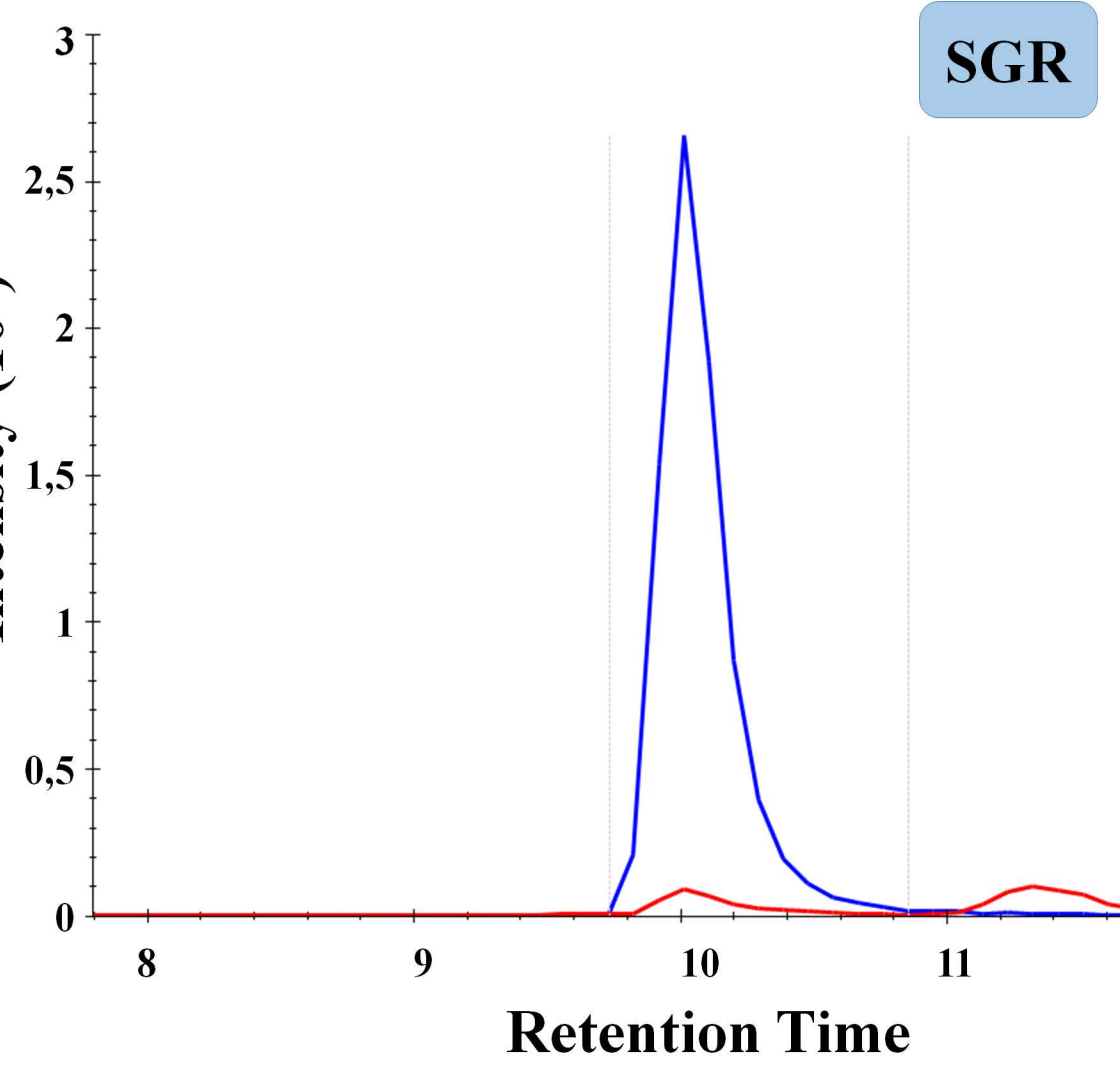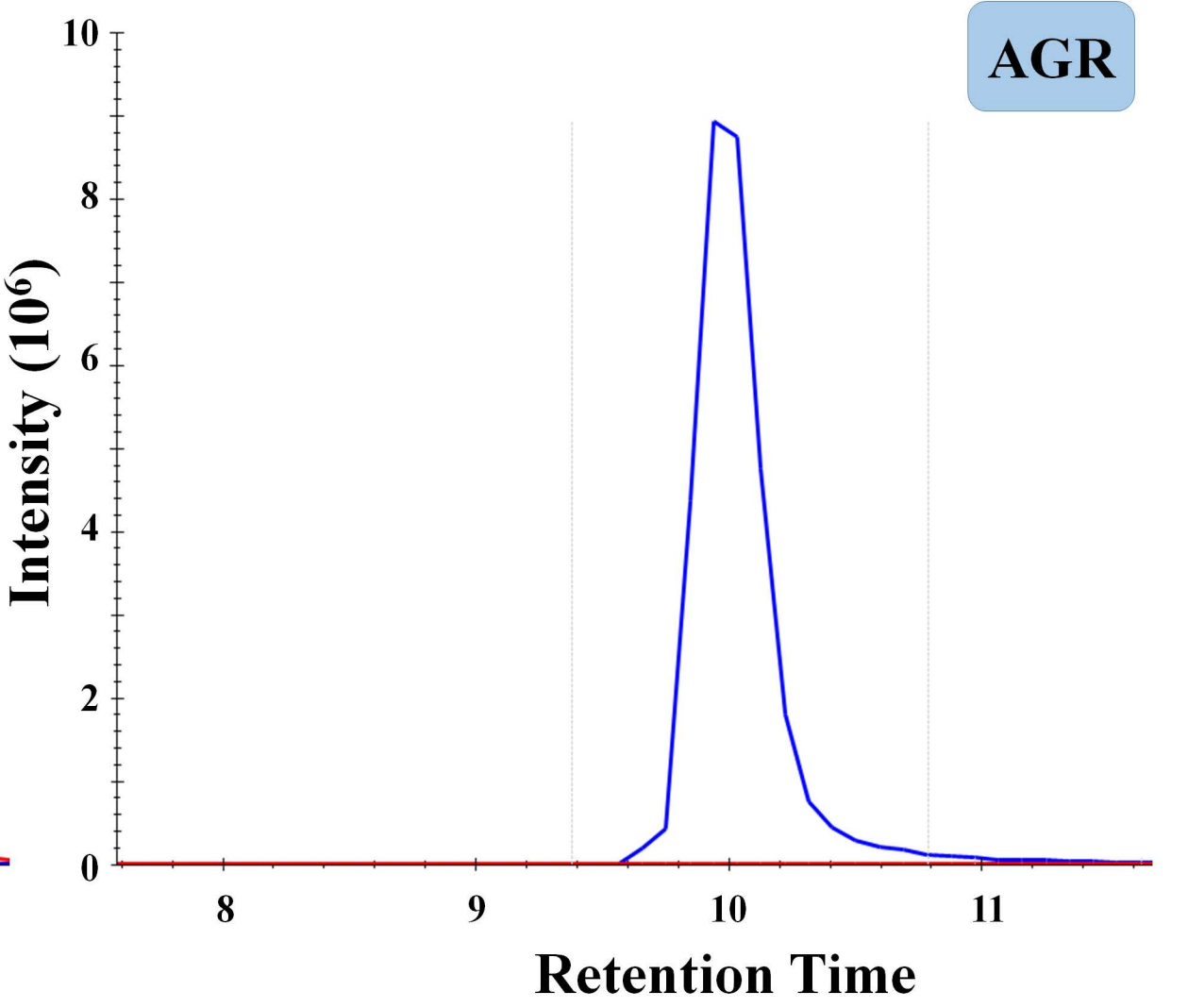

Supplement: Supplementary file 1 [file ijms-22-04233-s001.zip › Figure S1.pdf]

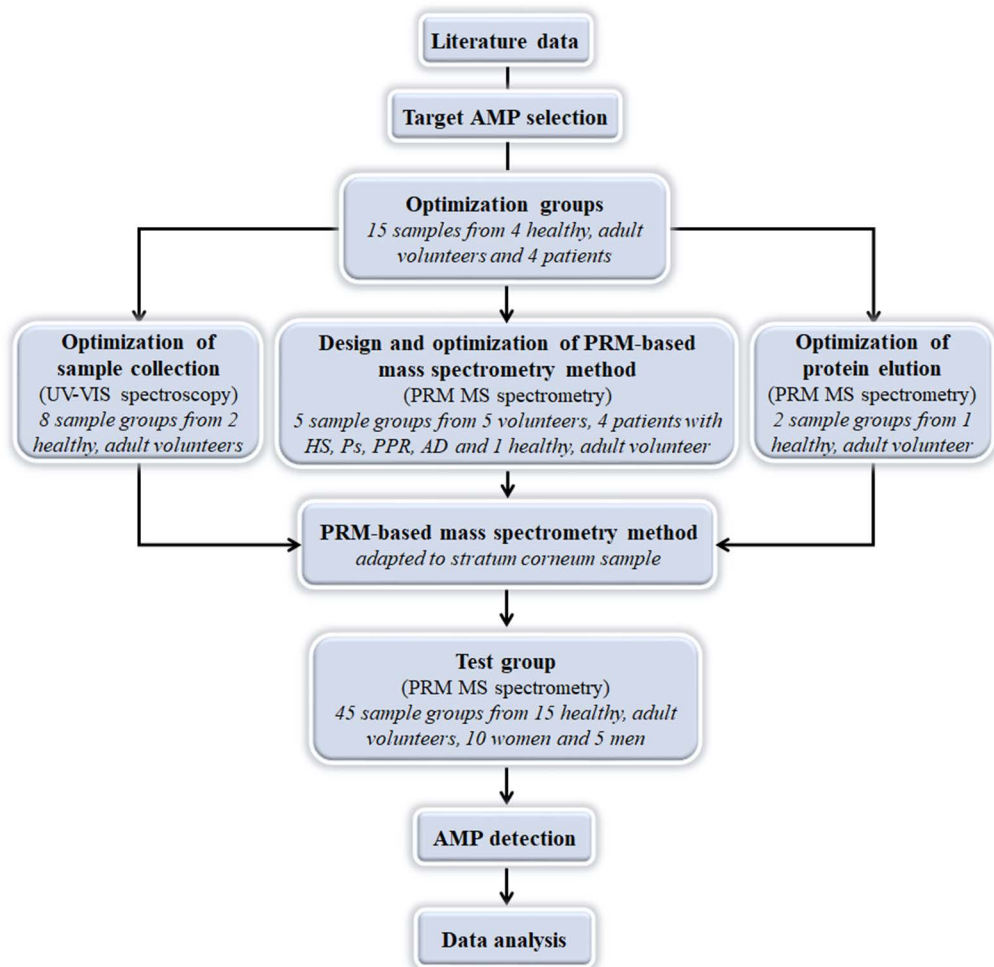

Supplement: Supplementary file 1 [file ijms-22-04233-s001.zip › Figure S2.pdf]
